# The green and sustainable radiology department

**DOI:** 10.1007/s00117-023-01189-6

**Published:** 2023-09-18

**Authors:** Jeffrey Mariampillai, Andrea Rockall, Christine Manuellian, Sara Cartwright, Samuel Taylor, Michael Deng, Sarah Sheard

**Affiliations:** 1 Hammersmith Hospital, Du Cane Road, W12 0HS London, UK; 2 Clinical Chair of Radiology, Department of Cancer and Surgery, Faculty of Medicine, Imperial College London, London, UK

**Keywords:** Sustainability, Greening radiology, Scope 3, Circular economy, Decarbonising, Nachhaltigkeit, Ökologische Radiologie, Scope-3-Standard, Kreislaufwirtschaft, Dekarbonisierung

## Abstract

As manmade climate change threatens the health of the planet, it is important that we understand and address the contribution of healthcare to global emissions. Medical imaging is a significant contributor to overall emissions. This article aims to highlight key issues and examples of sustainable practices, in order to empower radiologists to make a change within their department.

## Abstract (de)

Da der vom Menschen verursachte Klimawandel die Gesundheit des Planeten bedroht, ist es wichtig, den Anteil des Gesundheitswesens an den globalen Emissionen zu verstehen und anzugehen. Die medizinische Bildgebung stellt einen wesentlichen Mitverursacher der gesamten Emissionen dar. Ziel des vorliegenden Beitrags ist es, die zentralen Themen und Beispiele angewandter Nachhaltigkeit zu präsentieren, um Radiologen in die Lage zu versetzen, innerhalb ihrer Abteilung etwas zu verändern.

The health sectors of the United States, Australia, England, and Canada emit a combined 748 million metric tons of carbon dioxide annually. If the health sectors of these countries were a single independent nation, they would rank seventh in the world for greenhouse gas emissions [1]. Together with all healthcare sectors, radiology departments need to change their practices to become more sustainable. Healthcare is a polluting activity, and at the same time, climate change will have a negative impact on population health, disproportionately affecting vulnerable groups and therefore increasing health inequality. Our patients expect us to be environmentally conscious: 92% of UK patients found it important for their healthcare systems to operate more sustainably [2]. To effect change, the radiology sector needs to understand the sources of emissions from our activities and their proportional contributions, so that our mitigations have meaningful impact. Joining up policy change at governmental or organizational levels with activity at a local level is one of the greatest challenges to success. Radiologists and all imaging staff hold the key—via changes to the way they work in the department to using their influence with the organizations in which they work and from whom they purchase equipment. This article highlights some of the main ways in which radiology services generate emissions and discusses worthwhile evidence-based solutions which will allow radiologists to offer more sustainable services in the future.

## Scanner contribution

It is not surprising that the workhorses of the radiology department, MRI and CT scanners, account for significant carbon emissions: four MRI and three CT scanners alone were attributed to 4% of one hospital’s total energy consumption [2, 3]. A study from Basel, Switzerland, noted that the energy consumption of a CT scanner averaging 7904 patients a year was comparable to the usage of five four-person households. An MRI scanner averaging 4141 patients expends the same energy as 25.8 four-person households. One third of the total energy consumption of an MRI scanner is used for constant helium cooling and head cooling during the system off state. Two thirds of the overall energy consumption of a CT scanner is during its idling state, which is highly energy inefficient [3]. The study authors noted that efficient patient scheduling and flow through the scanner rooms would improve the carbon footprint by increasing the efficiency of room use and reducing energy lost whilst idle, energy that is not contributing to patient care. Energy-saving methods such as low-energy idle and system off states during nonproductive stages can reduce consumption by 25–33%. Innovative energy-conserving designs such as a power-save mode cycles the operations of the cold head compressor when the MRI is not subject to heat loads during active scanning, providing an additional 22–28% decrease in energy consumption [4].

Shortening MRI protocols with artificial intelligence algorithms such as the NYU FastMRI Dataset could result in decreased energy use in performing scans and reduce the need to install and operate additional scanners by increasing the throughput of existing MRI scanners [2].

## Optimal scheduling of investigations

Optimizing logistics with better scheduling of appointments is also an area to focus on. Virtual clinics, consultations via telephone, and video conferencing have recently taken off following the pandemic, resulting in reduction of the carbon emissions related to travelling to hospitals. The urology department at St. James Hospital, Dublin, Ireland, estimated a reduction of 6.07 t of carbon by conducting 72% of 1016 scheduled consultations via telephone in a 3-month period [5]. Imaging must be completed within the hospital; however, coordinating imaging appointments to coincide with outpatient clinics has practical, environmental, and financial benefits.

## Decision support: getting it right first time

In the same way radiologists justify the exposure of radiation to the patient they should also justify how their practice is sustainable. A study evaluating the American College of Radiology (ACR) Appropriateness Criteria, an evidence-based tool to aid clinicians in making appropriate imaging requests, identified that 48% of the listed patient conditions have other similar “usually appropriate” imaging modality choices which have different energy consumption levels [6]. Some modalities have significantly lower energy consumption compared to others: ultrasound trumps MRI, with 1.15 kg of carbon dioxide equivalents (CO_2_e) compared to 19.72 kg of CO_2_e per abdominal exam [7]. When devising protocols and guidelines, the energy consumption of each investigation should be considered and highlighted to the referring clinicians, particularly with regards to recommendations for the frequency of interval imaging or sufficient lower-energy modalities for follow-up imaging. More research is needed in areas where multiple repeat scans are currently favored: as patients live longer following successful treatment, research into appropriate follow-up intervals is an important area for investigation.

## Reporting stations and archiving data

The energy consumption of reporting stations may seem trivial when compared with the energy consumption during acquisition of images; however, reporting stations left on after working hours at. St Vincent’s Hospital, Dublin, Ireland, accounted for $ 7253 per year and CO_2_ emissions equivalent to the annual emissions of 10 passenger cars [8]. The University of Maryland Medical Center in the USA has shown 76.3% energy and cost savings (83,866.6 kWh and $ 9225.33, respectively) simply by shutting down workstations [9]. Shutting down the workstations will also reduce the energy consumption of heating, ventilation, and air conditioning (HVAC), as there are fewer reporting stations generating heat. Most modern computers have a standby mode where they will eventually switch off after a set number of hours idling. In a hypothetical scenario where 32 reporting stations switched off after 1 h rather than idling in standby for the default 4 h prior to switching off, energy consumption savings equivalent to 5 households or 3.8 passenger cars annually were estimated [10]. This is an example of where although automation can save some energy consumption, physically turning off appliances after usage is far superior. There is a very small peak in the consumption of energy when turning on workstations, but this is considered negligible compared to the overall savings of switching them off [11]. The only limiting factor is the time wasted in restarting computers; software for intelligent power management is an important development and implementation which would help with this aspect of power usage.

Data storage is often overlooked when considering the overall environmental impact of radiology. Data storage in general accounts for 2% of the worldwide consumption of electricity and contributes nearly the same amount of CO_2_ emissions as the airline industry [12]. As the quality of imaging increases, so does the size of the data stored. Data from the National Integrated Medical Imaging System (NIMIS), a centralized archiving system for most hospitals in Ireland, has shown an increase from 66 MB to 160 MB for the average CT study in the past 11 years [13]. A number of CT images saved are reconstructions of a single image series, which occupy a considerable amount of space. Modern picture archiving and communication system (PACS) software allows for multiplanar image reconstruction; therefore, only thin axial sections need to be maintained. As data increases at an exponential rate in the digital era, we should practice “digital temperance” with a shift in attitude towards restraint in production, use, and promotion of digital technologies whenever possible, ensuring the benefits outweigh the costs and financial or environmental impact [14].

There is UK government advice and legislation on the length of storage of primary care and hospital records [15]; however, there is very little advice on the longevity of data storage from imaging that is rising to exponential levels, with some reaching the levels of petabytes. It will be an ethical and contentious debate in the current setting of litigation and defensive medicine to bring about the appropriate and timely erasure of unnecessary imaging. There needs to be a public debate concerning long-term data storage. Patients and carers should be part of the decision-making process for developing guidance in data use for public good by way of data donation for research, or for agreements for data destruction after a suitable timeframe.

## Interventional radiology carbon footprint

The biggest contributors to the carbon footprint of interventional radiology (IR) have been investigated by a few groups. A study looking at the environmental impact of an IR department in New York, USA, demonstrated that in the average week it produced 23,500 kg of CO_2_. It would take 389 young trees 10 years to eliminate this amount of carbon. Almost half, 49%, of total CO_2_ production was attributed to HVAC; 57% of HVAC energy consumption occurred outside scheduled working hours, when few procedures were performed. The production and delivery of single-use surgical instruments accounted for 41% of the total CO_2_ production [16]. Reducing the use of HVAC outside normal operating hours by allowing the climate control system to drift within a wider range of temperatures and a reduction in the number of air changes has been suggested [16, 17]. Previously, it was falsely assumed that lowering or even turning off HVAC and then restarting the system to the required temperature would require even more energy than leaving it running. However, the American Society of Heating, Refrigerating, and Air-Conditioning Engineers advocate for a reduction in use during low-use operating room periods [17].

Plastics are a large component of single-use surgical instruments in the IR department. There is significant environmental harm caused by both the production and disposal of plastics. “Reduce, reuse, recycle” is often used when thinking about the sustainable waste management hierarchy, and this paradigm can also be applied when considering the use of plastic in healthcare. There is scope for reduction in the amount of plastic we use, especially in packaging. Several items in prepackaged surgical kits are unnecessary: 12 out of 40 disposable items in a prepackaged kit for tonsil surgery were considered unnecessary [18]. Wrapping sterile items using one-step sterile wrap carries no greater risk of bacterial contamination than double-wrap methods [19]. Reusing equipment is difficult given the risk of infection; however, some items are still considered single use even after sterilization mainly due to hypothetical risks with poor evidence, likely driven by the fear of prion disease. Most plastics used in healthcare are often incinerated. Less than 5% of plastic healthcare waste is recycled in the UK [18]. It has been suggested that 64% of operating theatre plastics could be recycled [18]. The barriers to medical plastic recycling include inadequate facilities or lack of engagement of staff. Plastics that are involved with medical waste must also undergo an additional step of steam sterilization before they can be recycled.

Imperial College Healthcare NHS Trust (ICHNT), a conglomerate of five hospitals with three imaging departments, carried out an 8‑week trial of reusable sterile gowns for use during interventional procedures in one hospital IR department as part of a drive to develop sustainable healthcare process. Subsequent findings demonstrated satisfaction in over 91% of staff. It is estimated that introducing this into all three imaging departments across ICHNT hospitals would reduce carbon emissions by an estimated 234,660 kg CO_2_ per year as well as resulting in an estimated financial saving of £ 27,131.

## Concepts of circular economy and sustainability in healthcare

The terms circular economy and sustainability are distinct concepts that are seemingly inherent in all environments. For the healthcare sector, the two terms serve a symbiotic relationship, that is to adopt a more sustainable approach to achieve a circular healthcare system. The goal is to shift from the traditional linear economic model—extraction of raw materials and manufacturing into consumer goods which are later disposed of as waste when the product reaches its end of life, which is resource intensive and wasteful—into a circular (closed-loop) economic model that aims to minimize resource consumption and waste generation. Traditionally, economic growth is attained through the linear model [20]. To achieve circular healthcare, the healthcare sector is required to provide products that are safe and sustainable by design, while reducing waste generation and exposure to harmful chemicals in its practices [21]. Through early communication and engagement with the supplier on sustainability agenda, the healthcare sector could increase its power of influence on its supply chain to set out clear and achievable emission reduction targets. This would minimize the impact on the environment by reducing greenhouse gas emissions, minimize resource consumption, and ensure effective waste stream management programs.

Manufacturing and installing new scanners requires substantial energy. After a set number of years, hardware needs to be replaced, but up to 95% of CT and MRI machine components can be used again [11]. Refurbishment, where most components stay in place, is even more sustainable than recycling decommissioned equipment. Manufacturers are also designing new scanners with upgradability in mind. Industry has shown commitment to sustainable practice: the Medical Imaging and Technology Alliance (MITA) has produced guidance and standards to help governmental organizations in developing directives and frameworks for the safe use of refurbished medical imaging equipment [22]. The refurbishment of medical imaging equipment has been an established practice for the past 20 years, accounting for 580 million USD in 2021 [23]. The circular economy of scanners is critical to future sustainability.

## Capturing scope 3 emissions

Scope 3 category emissions refer to all greenhouse gas emissions that are influenced by the activities of the organization but are not owned or controlled by the organization [24]. Due to the usage of contractors in the public sector, the size of scope 3 emissions is understood to be substantial compared to direct emissions. In 2019, the carbon footprint of the National Health Service (NHS) in the UK resulted in a total of 25 megatons of CO_2_e, of which 62% came from its supply chain [25]. This has been confirmed at the local level in a pilot study conducted at ICHNT. The study aimed to quantify full scope emissions for delivery of the diagnostic imaging service that is associated with a CT scanner. The results from the study showed that scope 3 emissions accounted for 69% of the total emissions, emphasizing how much net-zero targets in healthcare rely on decarbonization of the supply chain [26].

## Optimizing waste management

Inappropriate waste management in radiology departments can contribute to excessive carbon emissions and represents a large financial, unnecessary burden which can easily be reduced through a change in working practices. Improved waste management within one of the several separate CT departments at ICHNT dramatically reduced the amount of burns waste; estimated at saving just under £ 10,000 and 20,513 kg of CO_2_ per year, this equates to 6.5 flights from London to Perth [27].

Residual unwanted iodinated contrast media are often disposed of into the normal wastewater system or clinical waste stream [28]. GE Healthcare, supplier of contrast media, facilitate a collection service for excess contrast, introducing circularity into the system by avoiding pollution and reducing the need for raw iodine extraction [29]. This also keeps contrast bottles out of the high-emission clinical waste streams, allowing for recycling.

Re-educating staff on the use of the correct waste disposal streams within the imaging department such as general waste/clinical waste/burns waste/recycling waste can be embedded into training and induction processes, which will have the greatest impact within higher waste modalities such as CT (due to the modality’s high contrast usage) and interventional radiology. Foregrounding the wider impact both financially and environmentally can lead to a better understanding among colleagues of the effect of their actions and a more sustained change in behavior.

Clinical waste incineration can make use of a process which generates electricity (producing 0.347 t of negative carbon per ton of waste) and byproducts can be recycled or reused. Recycling programs even exist for old plain-film radiographs. This has the advantage of reducing the storage burden and may even be financially rewarding, as the silver they contain can be reclaimed.

Embedding waste management practices is essential for changes to persist after changes in staff. Changing default procurement codes to remove nonrecyclable stock is one method of embedding changes. Nonclinical examples are the most straightforward, such as removing polystyrene cups from purchasing and replacing with recyclable plastic cups, therefore reducing unsustainable waste. Ensuring access to appropriate waste streams, for example recycling facilities in staff and patient areas, is also important.

## Adopting change now

Change begins in the reporting room. We are all individually responsible for our own sustainable practices as healthcare providers. There are simple things we can do to encourage and promote sustainable practice among our own colleagues (Table 1).

**Table 1:** Sustainable practices that can be adopted now

Shutting down imaging equipment when not in use
Shutting down computers and monitors at the end of the day
Turning off heating and ventilation when not in use
Using motion-detector lights out of hours
Changing the habits of waste disposal, exchanging general waste bins for recycling bins
Minimizing the use of paper
Attending meetings virtually rather than crossing sites
Using sustainably sourced cleaning products
Removing single-use utensils and encouraging staff to bring their own cutlery

Auditing energy consumption of imaging departments during nonproductive hours where equipment is left on may highlight areas for improvement in energy efficiency. Heyes et al. produced a Python script to monitor the network status of multiple devices and equipment to determine if they were online during nonproductive hours: one CT, two PET/CT, one angiography suite, 16 PACS workstations, 20 printers, and six smart monitors were left on during nonproductive hours; the realized per-year energy savings accounted for 72,337 kWh, the energy consumption of 14 four-person households [30].

## Conclusion

Radiologists and radiographers are operators and consumers of healthcare equipment, and report to senior organizational management. In all these roles there is an important opportunity to effect change. In the department or clinic, we have seen how careful management of energy consumption, use of disposable items, and disposal of waste can have a large impact. The way we deliver our services influences the frequency and distance of staff and patient travel. Suppliers and manufacturers are already innovating, and environmental legislation will put them under additional pressure, but we need to use our purchase power to further reduce our supply chain emissions. As customers of manufacturers and suppliers, we can ask questions about supply chain sustainability in the form of embodied carbon, circularity of materials, or energy efficiency. There is some urgency to reducing carbon emissions across healthcare, so it is essential to share practice with colleagues and other specialties where there is overlap in activity. For issues managed further up the chain of command, such as hospital estates or energy supply, hearing the same request or concerns from multiple departments can increase the pressure to act. Let’s go!

## Declarations

For this article no studies with human participants or animals were performed by any of the authors. All studies mentioned were in accordance with the ethical standards indicated in each case.

The supplement containing this article is not sponsored by industry.
